# A Smart Alcoholmeter Sensor Based on Deep Learning Visual Perception

**DOI:** 10.3390/s22197394

**Published:** 2022-09-28

**Authors:** Savo D. Icagic, Goran S. Kvascev

**Affiliations:** 1 University of Belgrade, School of Electrical Engineering, Bulevar Kralja Aleksandra 73, 11120 Beograd, Serbia; 2 The Institute for Artificial Intelligence Research and Development of Serbia, Fruskogorska 1, 21000 Novi Sad, Serbia

**Keywords:** alcoholmeter, deep learning, convolutional neural network, automation, distillation, retrofitting

## Abstract

Process automation, in general, enables the enhancement of productivity, product quality, and consistency alongside other production metrics. Liquor production on an industrial scale also follows the automation trend. However, small and medium producers lag with equipment modernization due to the high costs of industrial equipment. One of the important sensors in automation equipment for distilleries is the alcohol concentration sensor used for fraction separation, process automation, and supervision. This paper proposes a novel low-cost approach to alcohol concentration sensing by employing deep learning on the visual perception of traditional alcoholmeter. For purposes of the training model, dataset acquisition apparatus is developed and a large dataset of labeled images of alcoholmeter readings is acquired. The problem of reading alcohol concentration from an alcoholometer image is treated as a regression and classification problem. Performances of both regression and classification models were investigated with Resnet18 as an architecture of choice. Both models achieved satisfying performance metrics demonstrating the feasibility of the proposed approaches. The proposed system implemented on Raspberry Pi with a camera can be integrated into new distillation equipment. Additionally, it can be used for retrofitting existing equipment due to its non-invasive nature of reading. The scope of use can be further expanded to the reading of other types of analog instruments simply by retraining the model.

## 1. Introduction

Liquor production is part of the tradition of almost all nations around the world. Famous examples of liquors with worldwide popularity are Scotch whisky and French cognac. Many more local national drinks exist such as rakija—fruit spirits manufactured in Balkan nations, Italian grappa made from grapes, Japanese sake made from fermented rice, etc.

The technology of liquor production has been perfected over centuries and involves two key technological processes—fermentation and distillation. Fermentation is common in the production of all alcoholic beverages. During alcoholic fermentation, yeasts convert sugar to ethanol, carbon dioxide, and other metabolic byproducts. Yeasts can typically survive up to 18% ethanol concentration, therefore the maximum concentration of ethanol in the fermented mash is around 18%. Liquors typically have an ethanol concentration of 30% or more, so fermented mash needs to be distilled in order to increase the concentration of ethanol in the beverage to the desired level. Distillation is the process of separating liquid mixture components through selective boiling and condensation. Mash is a complex mixture that dominantly contains water, ethanol, and other aromatic components. During distillation, each component has a different dynamic of transfer from the mash to the distillate. Some components of mash are desirable (pleasant aromatic components, ethanol) and some are undesirable (e.g., fusel oils) or even toxic (methanol). Therefore, distillate is typically separated into three fractions or “cuts“—heads, hearts, and tails. Heads come out first out of the still and contain a large concentration of methanol, aldehydes, and other alcohols [1]. Hearts is a desirable part of distillate; it has a good balance of pleasant aromatic compounds and a high concentration of ethanol. Tails are the fraction collected at the end of distillation; it contains undesirable fusel oils and low concentrations of ethanol [1].

There are two common methods for determining the moment of fraction separation. The first one is by organoleptic properties of distillate (by smelling and tasting samples of distillate). The other is by measuring the concentration of ethanol in distillate on the output of the still. The organoleptic method requires experienced and skilled experts, and it is vulnerable to the subjective criteria of each individual. Another important drawback of the organoleptic approach is poor scalability, i.e., if multiple stills have to be operated concurrently additional experts must be hired.

The ethanol concentration approach to fraction separation is based on the physical properties of output distillate [2]. Few empirical rules were established for separating fractions during the long history of liquor production. The common rule of thumb is to separate fractions when the alcohol concentration of distillate on the output still falls under some threshold. Threshold values are determined empirically and may vary concerning the quality of mash, type of fruit or grain in the mash, etc.

**Figure 1:**
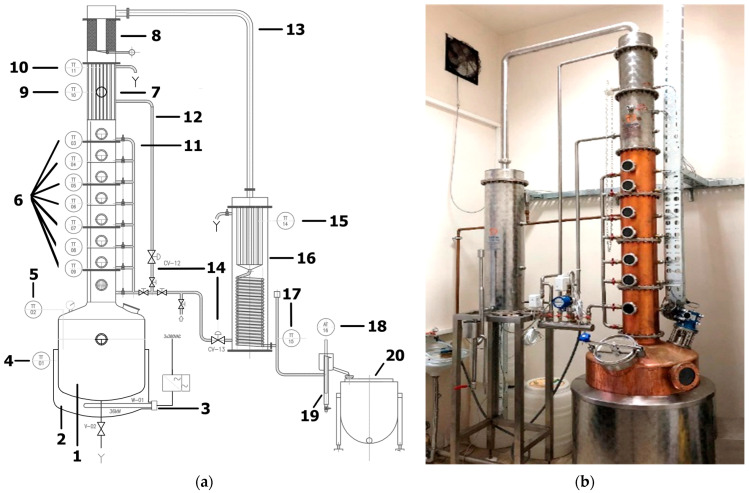
(**a**) Piping and instrumentation (P&ID) diagram of automated still with the following elements: 1. boiler with capacity of 100 L, 2. oil heating bath, 3. electric heater, 4. oil thermometer, 5. mash thermometer, 6. distillation column tray temperature thermometers, 7. dephlegmator, 8. catalyst, 9. dephlegmator coolant thermometer, 10. dephlegmator vapor thermometer, 11. water inlets for tray cleaning, 12. dephlegmator water supply pipe, 13. vapor, 14. water regulation valves, 15. condenser coolant thermometer, 16. combined cooler/condenser, 17. distillate thermometer, 18. alcoholmeter, 19. waste valve, 20. hearts cut container; (**b**) one realization of distillation unit.

The most important benefits of distillation process automation are the increase in consistency of liquor quality, power consumption reduction, and easier scaling of production. Several underlying processes of the distillation process can be automated. Thermal processes such as boiler and condenser temperature can be easily automated with industry-standard control algorithms such as PI regulation or bang-bang regulation. Today’s modern equipment for batch distillation often has some or all of these processes automated. One realization of the modern automated distillation unit is illustrated in Figure 1.

The fraction separation process is still mostly performed manually. The alcohol concentration fraction separation approach is objective and quantifiable criteria for fraction separation. Therefore, it is suitable for use in the automation of the distillation process. In order to close the control loop with the ethanol concentration approach, measurement of ethanol concentration is needed. There is a plethora of industrial-grade ethanol concentration sensors. However, the high price of industrial-grade sensors keeps them out of the reach of small and medium liquor producers and the conventional technique of measuring by alcoholmeter is a purely manual technique that requires a human operator.

This paper proposes a novel approach to ethanol concentration measurement. The proposed system combines the existing alcoholmeter and accompanying infrastructure with a deep learning-based measurement reading system. The system captures an image of an alcoholmeter which measures ethanol concentration and extracts measurement information from the image. A convolutional neural network (CNN) is used to extract ethanol concentration information from images. Two approaches to model architecture were analyzed based on the type of output layer—the regression and classification approach. The main motivation to use the regression model is the continual nature of its output which corresponds to the continual nature of ethanol concentration. The motivation to use the classification model comes from the discrete nature of alcoholmeter graduations. Both models were based on ResNet18 architecture [3], with output layer modification only. Due to specific application scenarios, models trained to start from pretrained ResNet18 models gave poor classification and regression performance. Therefore, models were randomly initialized and completely trained on the dataset generated for this paper.

Digitalization of analog instruments with gauge using vision-based systems has been demonstrated successfully many times since the vast majority of legacy instruments have gauge, such as aircraft instruments [4], water meter [5], energy meters [6], voltmeters [7], pressure gauges [8] and ammeters [9], power meters [10] etc.

Compared to typical gauges, even with fixed cameras, the alcoholmeter is not fixed in the frame; instead, it floats up or down and it can rotate, therefore making the automated alcoholmeter reading a significantly more complex problem. The image processing approach was used in the past for hydrometer alignment of measurement scale marks during calibration via immersion [11]. Exhaustive patents and the literature search did not yield the sensing approach proposed by this paper. 

Artificial intelligence (AI) techniques are increasingly used in the field of food and beverage sensorics. New AI-powered sensing techniques enhance the tracking quality of products during production and ensure both the quality and safety of finished products. 

Tonezzer et al. [12] developed a portable and inexpensive resistive gas sensor for distinguishing methanol from ethanol by using machine learning. Voss et al. [13] demonstrated alcohol detection in beers based on an electronic nose as an indirect method of ethanol concentration detection. Kuswandi et al. [14] developed a visual ethanol biosensor for halal verification of fermented beverage samples; however, this approach is not suitable for automation purposes due to its manual nature and only ethanol detection is achieved, not concentration measurement. Erfkamp et al. [15] developed a novel ethanol concentration sensor based on ethanol-sensitive hydrogels. The novel sensor shows promising performance as it demonstrated robustness with respect to a wide ethanol concentration range, pH variation, and salt concentration. The low technology readiness level (TRL) of this novel sensor prevents widespread use for now. Other techniques [16,17,18,19] for ethanol detection and concentration measurement are also the focus of many researchers.

This paper’s main goal is to demonstrate the ability of the deep learning-based system to digitalize visual alcoholmeter measurement, thus enabling the use of affordable and non-invasive sensing platforms in distilling automation. In addition to the proposed use in distillation automation, the system proposed in this paper can be reused for non-invasive reading of any human-readable instrument by retraining the model with an adequate dataset.

In Section 2, materials and methods, physical and practical aspects of traditional alcoholmeter are given first. Then, the proposed alcoholmeter reading system is described and the dataset acquisition setup apparatus and description are given. Image and label pre-processing are described afterward, and regression and classification models are defined at the end of Section 2. Results and dataset sample is presented in Section 3. Regression and classification performances are given visually and numerically. Section 4 represents the discussion. Results are discussed with respect to the initial proposal and methodology. Methodology improvements are also proposed based on the results. Section 5 is the conclusion. Final conclusions are given based on the previous discussion along with suggestions for further direction of research.

## 2. Materials and Methods

### 2.1. Traditional Alcoholmeter

**Figure 2:**
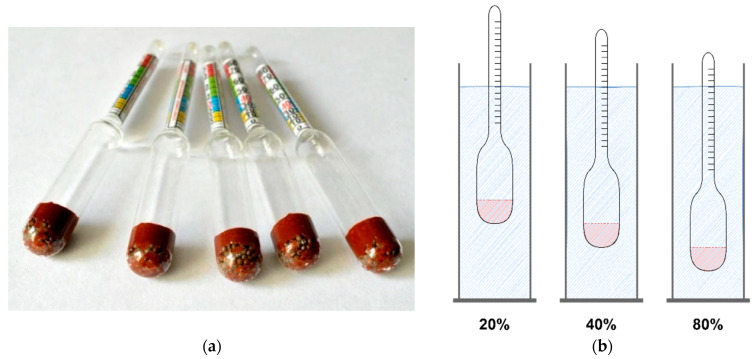
Traditional alcoholmeter illustration of: (**a**) various models with various measurement ranges; and (**b**) illustration of standard alcoholmeter measurement procedure and influence of alcohol concentration on alcoholmeter indication.

The traditional alcoholmeter is instrument used for measuring volumetric ethanol concentration in clear and sugar-free liquors. In fact, traditional alcoholmeter is hydrometer calibrated to measure volumetric alcohol concentration. Alcoholmeters used in beverage industry are often constructed in the form of sealed glass cylinders with wide bottom holding ballast and thin top cylinder which holds graduated scale. Figure 2a illustrates a few commercially available alcoholmeters of different sizes and measurement ranges.

In order to measure ethanol concentration in sample, alcoholmeter is placed in a tall container filled with sample fluid and left to float freely not touching the container walls, as illustrated in Figure 2b. When alcoholmeter stabilizes, wide bottom is submerged in liquid, and thinner upper part is partially submerged. Depending on alcohol concentration in sample, thin upper part of alcoholmeter will be submerged more or less in liquid. Alcohol concentration is indicated on graduated scale where liquid touches the stem of the alcoholmeter.

**Figure 3:**
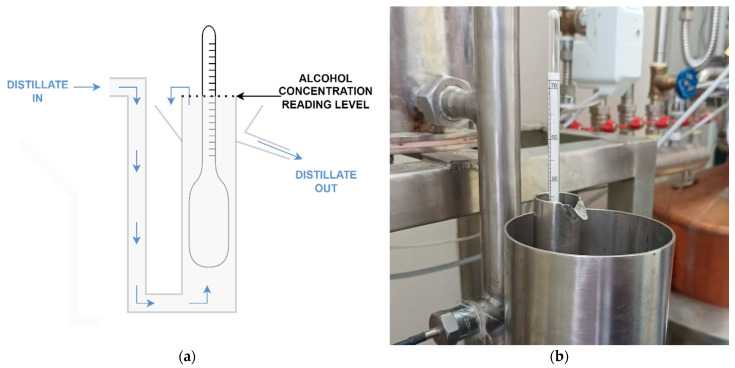
Structure of still parrot. (**a**) Illustration of parrot cross-section with distillate flow labeled with blue arrows and alcohol concentration reading level. Alcohol concentration is read from scale on alcoholmeter in place where it meets surface of the distillate; (**b**) one realization of parrot for measuring alcohol concentration during distillation.

In liquor production, alcohol concentration of output distillate flow is often continually measured or sampled periodically to track process progress and decide when to separate fractions. To perform continual measurement of alcohol concentration, alcoholmeter is placed in device called “parrot” Figure 3. “Parrot” is tall container connected to the output pipe still near its bottom. Distillate fills container and overflows it. Overflow distillate is collected and routed to the storage. Alcoholmeter is placed in the “parrot” which enables continual measurement of alcohol concentration in distillate stream. 

### 2.2. Alcoholmeter Reading System Description

**Figure 4:**
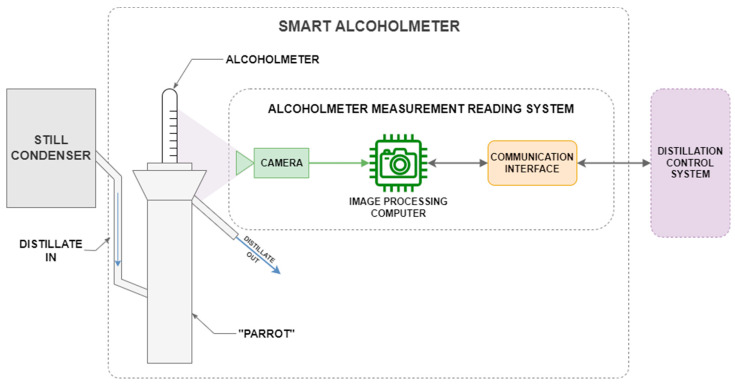
System diagram of complete alcohol concentration measurement system. It consists of parrot with alcoholmeter to measure alcohol concentration and alcoholmeter measurement reading system to digitalize measurement in real-time and supply it to distillation control or supervision system.

Smart alcoholmeter for distillation monitoring and/or control is based on traditional alcoholmeter and parrot with computer vision-based device for reading out measurements, as illustrated in Figure 4.

One sensing cycle of smart alcoholmeter consists of three consecutive steps. The first step is image acquisition. The command is issued to camera to acquire single image of alcoholmeter measurement indication. In the second step, the acquired image is fed into an image processing algorithm that extracts alcoholmeter reading from image. The third step is sending extracted measurements to the distillation control system for further use. System operates by continually repeating sensing cycle at preset frequency.

Various approaches could be used for alcoholmeter reading. Traditional image processing methods could be used as a part of alcoholmeter reading algorithm. However, they rely heavily on human to manually identify useful features and tune algorithm on given dataset. Considering large variety of commercially available alcoholmeters and their design differences, traditional image processing algorithm would need tuning for almost each one of them. 

In addition to traditional image processing methods, statistical pattern recognition techniques or statistical classification methods could be used for reading alcoholmeter. Due to large dimensions of images statistical models suffer from curse of dimensionality, i.e., they become very large and therefore inefficient. Dimension reduction, again, requires manual feature extraction making it specific for given dataset. Modern problem solving should not neglect traditional methods in favor of novel deep learning approach at all costs, as demonstrated by Liu et al. by using dynamic programming in solving state-of-the-art problems in autonomous driving planning [20].

However, the literature review indicates that, generally, deep neural networks achieve superior performance in both accuracy and robustness (e.g., change in lightning conditions, contrast, perspective); therefore, they are chosen as state-of-the-art approaches [4,5,6,7,8,9,10]. Convolutional neural networks represent de facto standard in the field of image processing and are chosen as an image processing model. A study by Chen et al. using CNNs for ship detection in coastal surveillance videos [21] is one of many examples which demonstrate usability and flexibility of CNNs in wide range of applications. Used deep neural network (DNN) models are described in subsection DNN Models.

To demonstrate proof of concept system, single board computer (SBC) was used as an image processing computer and a typical accessory camera was used for image acquisition. Due to good software support and good performance Raspberry Pi 4 B is chosen as an SBC device [22] and Raspberry Camera Module 2 is chosen for use as video camera. Hardware specifications of used SBC and camera are given in Table 1 and Table 2, respectively.

### 2.3. Dataset

#### 2.3.1. Acquisition Setup

**Figure 5:**
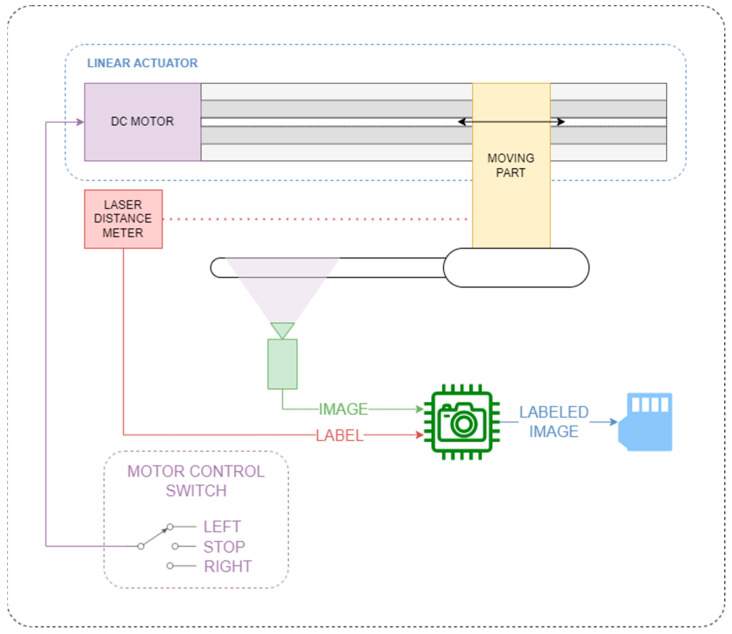
Dataset acquisition apparatus structural and functional illustration.

In general, training of CNNs requires a large dataset with at least a few thousand labeled images. Manual dataset acquisition and labeling is a tedious task and prone to human error. Therefore, semi-automated apparatus was constructed for dataset acquisition as shown in Figure 5.

Apparatus consists of three main components—linear actuator, laser distance sensor and image acquisition system. Image acquisition system consists of same SBC (Raspberry Pi 4 B) and video camera (Raspberry Pi Camera Module 2) intended for Smart Alcoholmeter System. Both SBC and camera are fixed on testbed. Laser distance sensor (Leuze ODSL 9/V6-450-S12) is fixed to testbed as well and pointed to the moving part of linear actuator in order to measure its linear position relative to the fixed testbed. Laser distance sensor has industrial standard analog output 0–10 V. Output voltage is attenuated 4 times and acquired by analog-to-digital converter (ADC). Important parameters of laser distance sensor and ADC are given in Table 3 and Table 4, respectively. Conventional alcoholmeter is attached to moving part of the linear actuator. Scale of alcoholmeter is placed in the field of view of camera. 

After mounting alcoholmeter onto the moving part of the apparatus, first step of dataset acquisition is running image acquisition and labeling script. The script captures image from camera and simultaneously polls ADC to obtain voltage measurement corresponding to distance of moving part, i.e., the alcoholmeter. Image and voltage are paired and stored in flash memory of SBC. Script repeats capture measure commands until predefined number of image–voltage pairs is acquired.

In the meantime, user manually controls linear actuator via control switch by setting direction of movement. The user controls direction of movement of actuator in such a way that whole alcoholmeter scale passes through camera field of view (FOV) in each pass.

In total, 15,098 samples are acquired. Dataset is split into three subsets—training, validation and test in ratio 70:10:20, respectively. Samples are randomly shuffled prior to splitting dataset.

#### 2.3.2. Image and Label Pre-Processing

In order to reduce complexity of future image processing, captured images are first cropped to the area of interest which predominantly contain alcoholmeter scale. Considering the fact that scale of alcoholmeter is black and white, and no information is coded in other colors, for sake of reducing computational complexity images are converted from RGB to grayscale thus reducing the size of input in CNN from 800 × 600 × 3 to 800 × 600 × 1. In total, input size to CNN is reduced from 800 × 600 × 3 to 370 × 191 × 1.

**Figure 6:**
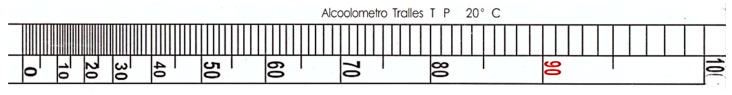
Flattened scale of one sample of commercially available alcoholmeter with obvious non-linear distribution of the graduations.

Labels acquired are linear function of spatial position of alcoholmeter in the image. However, graduations of alcoholmeter scale are not uniformly spatially distributed. Therefore, mapping from acquired labels to alcohol concentration labels is non-linear. With few exceptions, graduations of alcoholmeter scales are typically piecewise uniformly distributed. Most commonly, graduation of concentrations with values in multiples of 5% or 10% are non-uniformly distributed and called major graduation. Graduations between major graduations are approximately uniformly distributed, as shown on example of flattened scale of alcoholmeter on Figure 6 and called minor graduations. This feature is exploited to simplify mapping of measured voltages to alcohol concentrations.

Voltage–concentration to map measured voltages to ethanol concentrations piecewise uniformly to points $\left(c_{i},\;v_{i}\right)$ corresponding to major graduations are needed in order to map measured voltages to ethanol concentrations in piecewise uniform manner. They are obtained by manually finding images corresponding to each major graduation with concentration $c_{i}$ and difference between neighboring graduations $\Delta_{\%}$

(1) $$c_{i}=i\cdot \Delta_{\%},\;i=0,\;\ldots ,\;100/\Delta_{\%}$$

and pairing it with acquired voltage $v_{i}$. After getting voltage–concentration points for every major graduation, voltages of other samples in dataset can be mapped to concentrations by linear interpolation between major graduation points.

Voltage $v_{x}$ of sample $\left(c_{x},v_{x}\right)$ is compared with voltages $v_{i}$ of obtained major graduation points in order to determine nearest bounding major graduation points $\left(c_{k},v_{k}\right)$ and $\left(c_{k+1},\;v_{k+1}\right)$ i.e.,:

(2) $$v_{k}\leq v_{x}<v_{k+1}$$

therefore, concentration of sample $c_{x}$ must comply with

(3) $$c_{k}\leq c_{x}<c_{k+1}$$

Concentration label $c_{x}$ of sample is calculated by using linear interpolation between previously determined bounding major graduation points as following:

(4) $$c_{x}=c_{k}+\frac{c_{k+1}-c_{k}}{v_{k+1}-v_{k}}\cdot \left(v_{x}-v_{k}\right)$$

Finally, by repeating aforementioned procedure for each sample in dataset—all samples are labeled with alcohol concentration and ready for further use in training and testing. 

### 2.4. DNN Models

Resnet architecture is chosen as a starting point for DNN model development due to its efficient mitigation of vanishing gradient problem [3]. Output of the CNNs depends on the type of the output layer and it can be in various forms—most often categorical, continual value, or image. 

Considering continual nature of the ethanol concentration it is intuitive to expect such output from CNN, i.e., CNN should have regression output. Regression output should theoretically provide readout of alcoholmeter reading with sub-1% resolution due to its continual nature. 

The best accuracy of the measurement that can be achieved with absolute certainty by humans is the resolution of the alcoholmeter itself—most commonly not less than 1%. This perspective suggests that, instead of performing regression using CNN and getting continual value, discrete value of measurement with step of 1% or greater can be obtained by performing classification of the input image. Therefore, regression and classification approaches were put to the test as described in the following subsections.

#### 2.4.1. Regression

**Figure 7:**
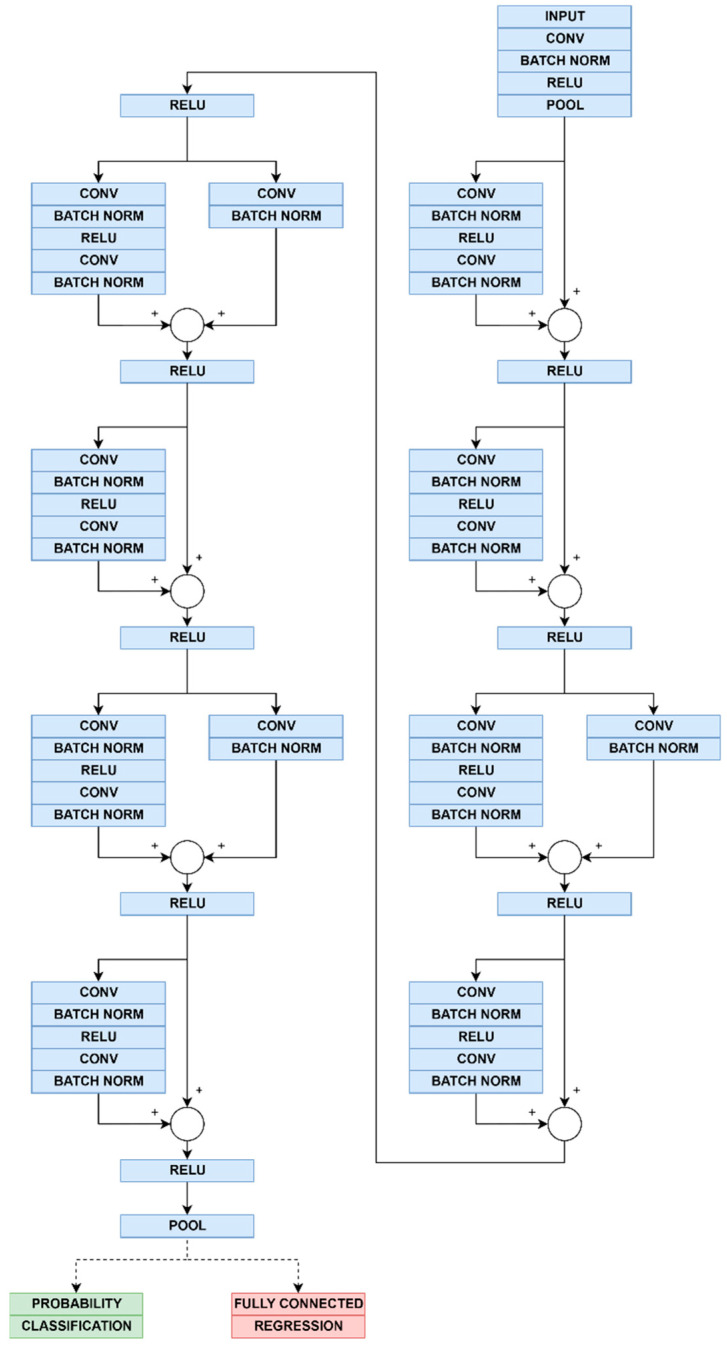
Proposed model CNN graph illustration. Resnet18 architecture of is common for both regression and classification models, except output layer and layer preceding it. Common part of model (blue layers), as well as difference between (a) regression model (red layers) and (b) classification model (green layers), are illustrated.

Original Resnet CNN architecture performs classification at the output layer, thus prompting slight alteration in the structure of the network. For this purpose, classification layer is replaced with regression layer, and probability layer is replaced with fully connected layer, as shown in Figure 7.

Common regression performance metrics are mean absolute error (MAE), bias (*μ*), root mean square error (RMSE), and coefficient of determination or R Square metric ($R^{2}$) defined by following expressions:

(5) $$MAE=\frac{1}{N}\Sigma_{i}\left|y_{i}-{\hat{y}}_{i}\right|$$

(6) $$\mu =\frac{1}{N}\Sigma_{i}\left(y_{i}-{\hat{y}}_{i}\right)$$

(7) $$RMSE=\sqrt{\frac{1}{N}\Sigma_{i}{\left(y_{i}-{\hat{y}}_{i}\right)}^{2}}$$

(8) $$R^{2}=1-\frac{\Sigma_{i}{\left(y_{i}-{\hat{y}}_{i}\right)}^{2}}{\Sigma_{i}{\left(y_{i}-\bar{y}\right)}^{2}}$$

(9) $$\bar{y}=\frac{1}{N}\Sigma_{i}y_{i}$$

where $y_{i}$ represents ground truth (alcohol concentration label), ${\hat{y}}_{i}$ represents prediction, i.e., reading of alcoholmeter by CNN, $\bar{y}$ is mean value of labels and N is number of samples.

#### 2.4.2. Classification

As Resnet18 architecture originally performs classification—no additional modifications were performed to the network, i.e., it is used in its original form, in addition to input dimension adaptation and number of output classes. Graph of Resnet18 network is given in Figure 7.

#### 2.4.3. Implementation

Training and performance evaluation is performed in MATLAB. Deep Learning toolbox has conventional Resnet18 neural network architecture built in. Default Resnet18 model is modified as described in previous subsection to adjust input image dimensions and type of output in order to perform regression or classification accordingly. Model is trained from scratch, pre-trained model is not used. Large number of simulations and experimental verification led to empirically determined training parameters given in Table 5.

## 3. Results

### 3.1. Dataset Samples

**Figure 8:**
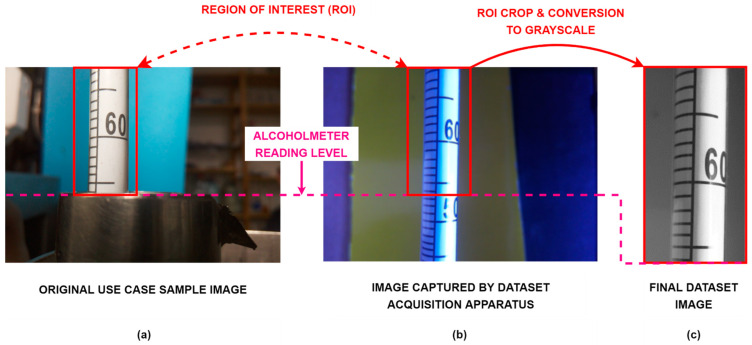
Illustration of dataset image generation and preprocessing procedure: (**a**) Original use case sample image is displayed with region of interest (ROI) labeled with red rectangle and alcoholmeter reading level labeled with pink dashed line; (**b**) Image captured by dataset acquisition apparatus together with ROI corresponding to original use case ROI and alcoholmeter reading level; (**c**) Final dataset image generated by cropping image acquired by apparatus to corresponding ROI and converting it to grayscale.

Size of originally acquired image is 800 × 600 × 3 as shown in Figure 8a with crop area marked with red rectangle. After image preprocessing sample takes its final form with size of 370 × 191 × 1 as shown in Figure 8c.

**Figure 9:**
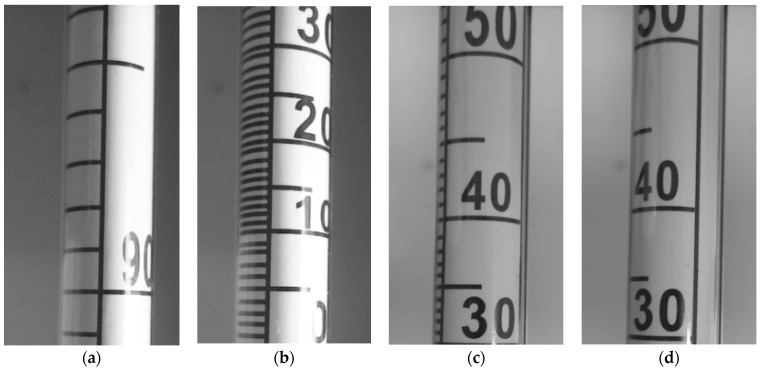
(**a**–**d**) Dataset samples of input images with different readings and orientations of alcoholmeter.

The camera capture trigger is not synchronized with the linear actuator position, so every image captured is different thus generating the desired variance of the dataset. An additional variance of the dataset is added by manually rotating the alcoholmeter axially and exposing different parts of the scale to the field of view (FOV) of camera and repeating process of dataset acquisition. To illustrate, a few samples of the dataset are shown in Figure 9.

**Figure 10:**
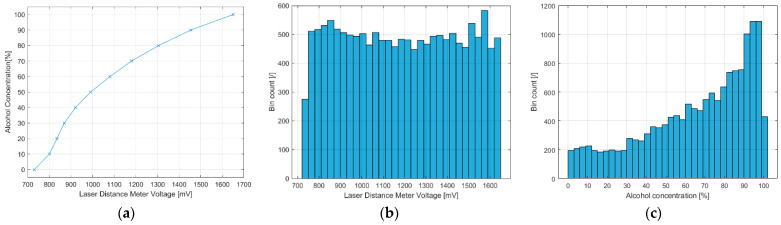
(**a**) Laser distance meter voltage to alcohol concentration mapping. X-marks represent 10% multiple voltage–concentration pairs and straight lines connecting them represent linearly interpolated values; dataset histograms in: (**b**) voltage domain and (**c**) alcohol concentration domain.

Voltage to alcohol concentration mapping of the acquired dataset is shown in Figure 10a. Voltage labeled histogram is uniform due to the uniform constant speed of linear actuator movement (Figure 10b). However, due to the non-linear mapping of voltage to alcohol concentration, the labeled dataset has a biased histogram toward higher alcohol concentrations due to greater spatial distance between graduations at higher alcohol concentrations (Figure 10c). 

### 3.2. DNN Training Performance

#### 3.2.1. Regression Performance

For test dataset regression model performance metrics MAE, RMSE, and $R^{2}$ are presented in Table 6.

**Figure 11:**
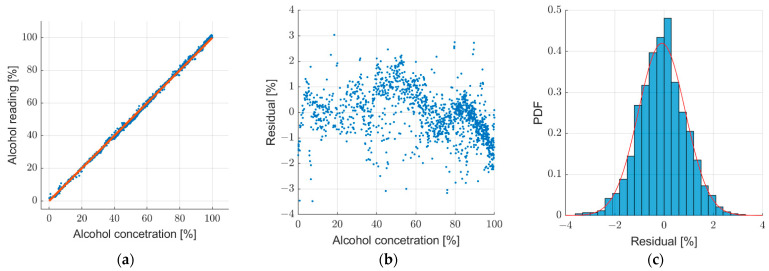
Regression model prediction on test dataset results: (**a**) scatter plot of alcohol reading versus alcohol concentration label (blue dots) and ideal sensor characteristic (red line); (**b**) scatter plot of residuals with respect to alcohol concentration labels; (**c**) normalized histogram of residuals (blue bins) and fit of Gaussian probability density function (red curve).

The visualization of trained regression model on test dataset is shown in Figure 11. In Figure 11a, the scatter plot of the alcoholmeter reading versus the sample label (blue dots) is shown together with the ideal linear transfer characteristic of the sensor (red line). Residuals $e_{i}=y_{i}-{\hat{y}}_{i}$ on the test dataset can be seen in Figure 11b. The inference time of the regression model on the one image sample is on average circa 500 milliseconds. 

#### 3.2.2. Classification Performance

**Figure 12:**
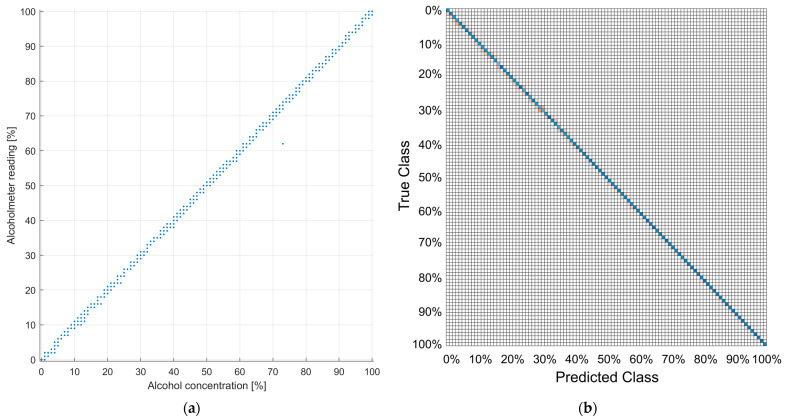
Classification results: (**a**) scatter plot of alcoholmeter readings versus ground truth alcohol concentration; (**b**) confusion matrix plot—blue fields represent true positives (where prediction made by model matches ground truth) and red fields represent misclassified samples.

Visualization of classification performance on the test dataset is given in Figure 12. The scatter plot of the alcoholmeter reading vs. alcohol concentration label is displayed in Figure 12a. Performance is additionally illustrated with the confusion matrix in Figure 12b. A Scatter plot enables better outlier visualization than a confusion matrix. On the other hand, the confusion matrix gives better insight into the relative ratios of true positive and misclassified samples. The inference time of the classification model on one image sample is on average circa 500 milliseconds.

## 4. Discussion

The dataset acquisition apparatus produces uniformly distributed samples (Figure 10b) with voltage labels due to the constant speed of the linear actuator. However, due to the non-linear mapping of voltage to alcohol concentration (Figure 10a), skewness appears in the alcohol concentration domain of labels. One aspect of apparatus improvement could be automatic control of the linear actuator, eliminating the need for manual control. With closed-loop speed/position control of the actuator, uniform distribution of samples could be achieved in the alcohol concentration domain of labels. Further improvement of the apparatus can be made by introducing an additional rotary actuator for the alcoholmeter to eliminate manual rotation and generate a more continuous dataset.

Regression model evaluation on the test dataset yielded an excellent performance as shown in Figure 11. Readings produced by the model are densely grouped near the ideal reading characteristic of Figure 11a. This is backed by the fact that RMSE and MAE have values of less than 1% (Table 6). Reading error is additionally visualized with the residual plot (Figure 11b) which suggests that slight variation of reading error with respect to alcohol concentration. Further analysis of reading error indicates Gaussian distribution of reading error (Figure 11c) with bias less than 0.1% (Table 6) which can be compensated. A value of 0.9988 for the R-squared metric additionally testifies to excellent reading performance.

Classification model evaluation on the test dataset also yielded more than satisfactory performance as shown in Figure 12. By classifying rather than fitting the regression model output, the model is quantized to 1% quant. Accuracy of ±1% is effectively achieved as shown in the scatter plot (Figure 12a) and confusion matrix plot (Figure 12b), with only a handful of outliers. With the reading rate being significantly larger (circa 500 ms per sample) than the rate of change in alcohol concentration, outlier rejection can be easily implemented by filtering the model’s output with a simple median filter.

## 5. Conclusions

The paper presents a novel approach to the digital measuring of alcohol concentration using a traditional alcoholmeter and deep learning visual perception-based system for measurement reading. Alcohol concentration measurement is important in the automation of the distillation of liquor. Available alcohol concentration sensors for automation purposes exist; however, their high price keeps them out of the reach of small and medium producers. Approach to alcohol concentration sensing proposed by this paper addresses this problem in two ways. Firstly, it presents a low-cost solution by demonstrating the use of affordable shelf hardware. Secondly, it represents a non-invasive solution with no need for structural modification of any kind to existing distillation equipment.

The core problem of reading alcoholmeter measurement was treated both as a regression and classification deep learning problem. Resnet18 convolutional neural network architecture proved to be a good choice for addressing the core problem as it demonstrated excellent performance in both regression and classification approaches. Considering the satisfactory performance of the proposed network architectures for automation purposes, no other network architectures were tested.

The density of liquid depends on its temperature. Therefore, measurement correction must be performed if the temperature of the sample is not equal to the temperature of alcoholmeter calibration. Temperature compensation is often linear function which makes temperature compensation simple. One aspect of future improvements of alcoholmeter measurement accuracy is the implementation of automatic temperature compensation.

The proposed system can be integrated as a part of new automated distillation equipment, and due to its non-invasive nature, it can be used in the retrofitting and modernization of existing distillery equipment with minimal modifications to existing infrastructure.

The scope of application of the proposed system can be further expanded to reading other types of instruments used in the industry by simply training models on the appropriate dataset. Significant cost reduction in retrofitting or modernization of existing control and supervision systems can be achieved with the proposed approach of instrument digitalization, as it minimizes downtime during integration and eliminates the need for buying new digital sensors.

## 6. Patents

Patent application P-2022/0809 under the title System and method for digitalization of alcoholmeter measurements based on artificial intelligence is applied on 25 August 2022.

## Figures and Tables

**Table 1:** Table of single board computer specifications [23].

Single Board Computer Data	Value
Manufacturer	Raspberry Pi Foundation, Cambridge, England, UK
Model	Raspberry Pi 4 model B
CPU	Broadcom BCM2711, Quad core Cortex-A72 (ARM v8) 64-bit SoC @ 1.5 GHz
RAM	4 GB LPDDR4-3200 SDRAM
Camera Interface	2-lane MIPI CSI camera port
Power requirements	5 V 3 A

**Table 2:** Table of camera specifications [24].

Camera Data	Value
Manufacturer	Raspberry Pi Foundation, Cambridge, England, UK
Model	Raspberry Pi Camera Module 2
Sensor	Sony IMX219
Still resolution	8 megapixels
Sensor resolution	3280 × 2464 pixels
Sensor image area	3.68 × 2.76 mm (4.6 mm diagonal)
Pixel size	1.12 µm × 1.12 µm
Optical size	1/4″
Horizontal Field of View	62.2 degrees
Vertical Field of View	48.8 degrees
Focal length	3.04 mm
Communication interface	CSI

**Table 3:** Table of laser distance sensor parameters [25].

Laser Distance Sensor Data	Value	Unit
Manufacturer	Leuze electronic GmbH + Co. KG, Owen, Germany	/
Model	ODSL 9/V6-450-S12	/
Range	50–450	mm
Resolution	0.1	mm
Accuracy	1	%
Repeatability	0.5	%

**Table 4:** Table of analog-to-digital converter [26].

Analog-to-Digital Converter Parameters	Value	Unit
Manufacturer	Microchip Technology Inc; Chandler, AZ, USA	/
Model	MCP3564R	/
Resolution	24	bit
SINAD	106.7	dB
RMS Effective Resolution (max)	23.3	bit

**Table 5:** Model training parameters table.

Parameter Name	Value
Optimization method	ADAM
Mini-batch size	32
Max Epochs	10
Initial Learn Rate	5 × 10^−4^
Learn Rate Drop Factor	0.1
Learn Rate Drop Period	20

**Table 6:** Regression model statistical performance.

Statistical Parameter	Value on Test Dataset
Mean absolute error (MAE)	0.7493
Bias (*μ*)	−0.0877
Root mean square error (RMSE)	0.9531
R-squared (*R*^2^)	0.9988

## Data Availability

All the data are in the manuscript.
